# Reducing computational demands of restricted maximum likelihood estimation with genomic relationship matrices

**DOI:** 10.1186/s12711-023-00781-7

**Published:** 2023-01-25

**Authors:** Karin Meyer

**Affiliations:** AGBU, A Joint Venture of NSW Department of Primary Industries and University of New England, Armidale, NSW 2351 Australia

## Abstract

Restricted maximum likelihood estimation of genetic parameters accounting for genomic relationships has been reported to impose computational burdens which typically are many times higher than those of corresponding analyses considering pedigree based relationships only. This can be attributed to the dense nature of genomic relationship matrices and their inverses. We outline a reparameterisation of the multivariate linear mixed model to principal components and its effects on the sparsity pattern of the pertaining coefficient matrix in the mixed model equations. Using two data sets we demonstrate that this can dramatically reduce the computing time per iterate of the widely used ‘average information’ algorithm for restricted maximum likelihood. This is primarily due to the fact that on the principal component scale, the first derivatives of the coefficient matrix with respect to the parameters modelling genetic covariances between traits are independent of the relationship matrix between individuals, i.e. are not afflicted by a multitude of genomic relationships.

## Background

With the increasing availability of genomic information for livestock genetic evaluation, incorporating such information has become a routine procedure. The most common method in use is that of single-step genomic best linear unbiased prediction fitting a breeding value model, abbreviated to ssGBLUP. Replacing the pedigree-based inverse of the numerator relationship matrix with its counterpart which combines pedigree and genomic information [1], it is a conceptually simple extension of classic prediction models. This has been exploited in adapting existing software to single-step analyses both for ssGBLUP and the estimation of genetic parameters via restricted maximum likelihood (REML) under such a model, which is referred to as ssGREML.

Reviewing the status of genomic evaluation, Misztal et al. [2] advocated inclusion of genomic relationships when estimating genetic parameters to counteract bias due to genomic selection. The authors also recommended more frequent re-estimation as genetic variances appeared to change more quickly, but warned about the associated computational burden. Contrasting computing times for pedigree-based REML with ssGREML for a data set with 15,723 genotyped animals, Masuda et al. [3] reported 100-fold increases for the latter. Suggestions that aimed at reducing computational demands of ssGREML analyses included approximation of the inverse of the genomic relationship matrix by its APY (algorithm proven and young [4]) form, coupled with truncation of long pedigrees [5]. Other studies demonstrated the utility of a REML algorithm based on the inverse of the phenotypic covariance matrix rather than the mixed model equations (MME), e.g. Lee and van der Werf [6].

Previously, there has been interest in parameterising multivariate REML analyses to fit genetic principal components rather than the standard trait effects. This was motivated by the possibilities for dimension reduction and for estimating covariance matrices with reduced rank or a factor-analytic structure [7–10]. When fitted at full rank, the standard multivariate (MV) and principal components (PC) parameterisation yield equivalent models but the pertaining MME differ in sparsity of the coefficient matrix. Limited comparisons (unpublished) of the two models for pedigree-based REML showed negligible differences in computational requirements for two reasons. First, due to ‘fill-in’, sparsity levels of the corresponding Cholesky factors of the coefficient matrices and thus operation counts to factor and invert them were comparable. Second, the inverse of the numerator relationship matrix was very sparse, so that the reduction in calculations proportional to the number of non-zero elements in the coefficient matrix for PC had little impact on overall computing times. Conversely, this suggests that for ssGREML–with denser inverse relationship matrices—gains that are possible by fitting the PC model may be more substantial.

The so-called ‘average information’ (AI-REML) algorithm [11–14] is the preferred algorithm to locate the maximum of the likelihood function for many REML analyses as it uses both first and second derivatives of the likelihood which tends to facilitate good convergence rates. We examine its computational requirements for ssGREML for the two alternative parameterisations for two data sets with moderate numbers of genotyped individuals, showing that the PC parameterisation can be highly effective in reducing computing times.

## Equivalent models

Consider a multivariate linear mixed model for *q* traits:

1 $$\mathbf{y}= \mathbf{X} {\boldsymbol{\upbeta}} + \mathbf{Zu} + \mathbf{e} ,$$

with **y**, ${\varvec{\upbeta}}$, **u** and **e** denoting the vectors of observations, fixed effects, animals’ additive genetic effects and residuals and **X** and **Z** the pertaining design matrices, where **y** is assumed to have a multivariate normal distribution and **X** is assumed to have full rank. Let both **y** and **u** be ordered by traits within individuals. This gives ${\text{Var}}(\mathbf{e}) = \mathbf{R}$ which is block-diagonal for animals with blocks equal to submatrices of ${\varvec{\Sigma}_{e}}$ corresponding to the (subset of) traits recorded, with ${\varvec{\Sigma}_{e}}$ the $q \times q$ matrix of residual covariances between traits. Furthermore, ${\text{Var}}\left(\mathbf{u} \right) = \mathbf{H} \otimes {\varvec{\Sigma}_{u}}$, with **H** the relationship matrix between animals, ${\varvec{\Sigma}_{u}}$ the $q \times q$ genetic covariance matrix between traits and ‘$\otimes$’ denoting the Kronecker product. Among other options, **H** can represent the pedigree-based numerator relationship matrix, the genomic relationship matrix or the joint relationship matrix between genotyped and non-genotyped individuals [1].

The pertaining mixed model equations (MME) equations are then:

2 $$\left[ \begin{array}{cc} \mathbf{X}^{\prime} \mathbf{R}^{-1}\mathbf{X} & \mathbf{X}^{\prime} \mathbf{R}^{-1}\mathbf{Z}\\ \mathbf{Z}^{\prime} \mathbf{R}^{-1}\mathbf{X} & \mathbf{Z}^{\prime} \mathbf{R}^{-1}\mathbf{Z} + \mathbf{H}^{-1}\otimes {\varvec{\Sigma}}_{u}^{-1} \end{array} \right] \left[\begin{array}{ll} \hat{{\varvec{\upbeta}}} \\ \hat{\mathbf{u}} \end{array} \right] = \left[ \begin{array} {ll} \mathbf{X}^{\prime} \mathbf{R}^{-1}\mathbf{y} \\ \mathbf{Z}^{\prime} \mathbf{R}^{-1}\mathbf{y} \end{array} \right].$$

Let **C**, partitioned as in Eq. (2), denote the coefficient matrix in the MME:

3 $$\mathbf{C} = \left[\begin{array} {cc} \mathbf{X}^{\prime} \mathbf{R}^{-1}\mathbf{X} & \mathbf{X}^{\prime} \mathbf{R}^{-1}\mathbf{Z}\\ \mathbf{Z}^{\prime} \mathbf{R}^{-1}\mathbf{X} & \mathbf{Z}^{\prime} \mathbf{R}^{-1}\mathbf{Z} + \mathbf{H}^{-1}\otimes {\varvec{\Sigma}}_{u}^{-1} \end{array} \right]= \left[\begin{array}{cc} \mathbf{C}_{\beta \beta} & \mathbf{C}_{\beta u} \\ \mathbf{C}_{\beta u}^{\prime} & \mathbf{C}_{uu}\end{array} \right].$$

Each of the four submatrices has non-zero elements contributed by the data, i.e. arising from (weighted) products of the design matrices **X** and **Z**. For $\mathbf{C}_{uu}$, $\mathbf{Z}^{\prime} \mathbf{R}^{-1}\mathbf{Z}$ is sparse, consisting of diagonal blocks of size $q \times q$ for *N* individuals. For traits that are not recorded, corresponding rows and columns (of the diagonal blocks) have zero elements. The second part of $\mathbf{C}_{uu}$, $\mathbf{H}^{-1} \otimes {\varvec{\Sigma}}_{u}^{-1}$, contributes a $q \times q$ block for each non-zero element of $\mathbf{H}^{-1}$.

An alternative formulation is obtained by rewriting Eq. (1) as [10, 15]:

4 $$\mathbf{y}= \mathbf{X} {\varvec{\upbeta}} + \mathbf{Z} \left ( \mathbf{I}_N \otimes \mathbf{Q} \right ) \left ( \mathbf{I}_N \otimes \mathbf{Q}^{-1} \right ) \mathbf{u} + \mathbf{e}= \mathbf{X} {\varvec{\upbeta}} + \mathbf{Z}^{\star}\mathbf{u}^{\star}+ \mathbf{e} ,$$

with $\mathbf{I}_N$ denoting an identity matrix of size *N*, $\mathbf{Z}^{\star}= \mathbf{Z} \left (\mathbf{I}_N \otimes \mathbf{Q}\right )$ and $\mathbf{u}^{\star}= \left ( \mathbf{I}_N \otimes \mathbf{Q}^{-1} \right ) \mathbf{u}$. This gives ${\text{Var}}\left( \mathbf{u}^{\star}\right) = \mathbf{H} \otimes \mathbf{Q}^{-1}{\varvec{\Sigma}_{u}}\mathbf{Q}^{-T}$ and it follows that $\mathbf{Z} {\text{Var}}\left( \mathbf{u} \right) \mathbf{Z}^{\prime} = \mathbf{Z}^{\star}{\text{Var}}\left( \mathbf{u}^{\star}\right) \mathbf{Z}^{\star}{^{\prime}}$. For computational efficiency, we can choose **Q** so that $\mathbf{Q}^{-1}{\varvec{\Sigma}_{u}}\mathbf{Q}^{-T} = \mathbf{I}_q$. This is an equivalent model to Eq. (1) with the coefficient matrix in the pertaining MME:

5 $$\mathbf{C}^{\star}= \left[\begin{array}{cc} \mathbf{X}^{\prime} \mathbf{R}^{-1}\mathbf{X} & \mathbf{X}^{\prime} \mathbf{R}^{-1}\mathbf{Z}^{\star}\\ \mathbf{Z}^{\star}{^{\prime}} \mathbf{R}^{-1}\mathbf{X} & \mathbf{Z}^{\star}{^{\prime}} \mathbf{R}^{-1}\mathbf{Z}^{\star}+ \mathbf{H}^{-1}\otimes \mathbf{I}_q \end{array}\right] = \left[\begin{array}{ll} \mathbf{C}_{\beta \beta} & \mathbf{C}_{\beta u}^{\star} \\ {\mathbf{C}_{\beta u}^{\star}}^{\prime} & \mathbf{C}_{uu}^{\star}\end{array}\right] .$$

The transformation can be thought of as transferring genetic links between traits from the part due to the covariance of the random effect to the ‘data part’ of the coefficient matrix. For single records per trait, each row of **Z** typically has a single non-zero element of unity. In contrast, $\mathbf{Z}^{\star}$ contains up to *q* non-zero coefficients per row, positioned in the columns representing the respective animals’ genetic effects for the *q* traits. Thus, the first part of $\mathbf{C}_{uu}^{\star}$ is again block-diagonal for individuals, though — compared to $\mathbf{C}_{uu}$ – some additional non-zero elements arise for missing records. Similarly, some additional non-zero elements can be generated in $\mathbf{C}_{\beta u}^{\star}$ compared to $\mathbf{C}_{\beta u}$. The second component ($\mathbf{H}^{-1}\otimes \mathbf{I}_q$) of $\mathbf{C}_{uu}^{\star}$, however, is substantially sparser than its counterpart in $\mathbf{C}_{uu}$, the more so the higher *q* and the denser $\mathbf{H}^{-1}$. Here each non-zero off-diagonal element of $\mathbf{H}^{-1}$ contributes only *q* additional non-zero coefficients in Eq. (5) compared to $q^2$ in the above Eq. (3).

Suitable choices for **Q** arise from the eigen-decomposition of the genetic covariance matrix, ${\varvec{\Sigma}_{u}} = \mathbf{E} {\boldsymbol{\Lambda}} \mathbf{E}^{\prime}$. For **Q** equal to the matrix of eigenvectors, **E**, $\mathbf{u}^{\star}$ would be equal to the vector of genetic principal component scores, which can be scaled to variances of unity by setting $\mathbf{Q} = \mathbf{E} {\boldsymbol{\Lambda}}^{1/2}$ (with ${\boldsymbol{\Lambda}}$ the diagonal matrix of eigenvalues). Thus, we refer to Eq. (4) as the ‘principal components’ model. In general, this form of **Q** has $q^2$ non-zero elements. This can be reduced to $q(q+1)/2$ by an orthogonal transformation to lower triangular form or, more generally, by setting $\mathbf{Q}$ to be a Cholesky factor of ${\varvec{\Sigma}_{u}}$. Furthermore, the latter integrates well with REML implementations which often parameterise to estimate the elements of the Cholesky factor of covariance matrices rather than the covariance components directly.

REML estimation based on the transformation to principal components is implemented in our mixed model package $\mathbb {WOMBAT}$ [16, 17] for selected models of analysis.

## Average information REML

In general, REML estimation of variance components represents a non-linear optimisation problem that is solved iteratively. Let $\log {\varvec{\mathcal {L}}}$ be the REML likelihood on the logarithmic scale and let ${\varvec{\uptheta}}$, with elements $\theta _i$, denote the vector of parameters to be estimated. The basic Newton(-Raphson) algorithm to update estimates from iterate *k* to iterate $k+1$ is then:

6 $${\varvec{\uptheta}}^{k+1}= {\varvec{\uptheta}}^{k} - \omega \mathbf{I}\left ({\varvec{\uptheta}}^{k}\right )^{-1} \mathbf{s}\left ( {\varvec{\uptheta}}^{k} \right ),$$

where $0 < \omega \le 1$ is a scalar to modify step sizes where necessary to avoid ‘overshooting’. Terms $\mathbf{s}\left ( {\varvec{\uptheta}}^{k} \right )$ and $\mathbf{I}\left ({\varvec{\uptheta}}^{k}\right )$ represent the gradient vector and information matrix, respectively, both evaluated at ${\varvec{\uptheta}}^{k}$. These have elements equal to the first and second partial derivatives of $\log {\varvec{\mathcal {L}}}$ with respect to the parameters to be estimated, $\theta _i$. Let $\theta _{ui}$ and $\theta _{ei}$ denote parameters modelling ${\varvec{\Sigma}_{u}}$ and ${\varvec{\Sigma}_{e}}$, respectively. For the AI-REML algorithm, the second derivatives of $\log {\varvec{\mathcal {L}}}$ are replaced with their counterparts considering the ‘data part’ of the likelihood only. For $\mathbf{V} = {\text{Var}}(\mathbf{y})$ linear in the vector of parameters, these are equal to the average of observed and expected information [11] —  hence its name.

Details of the AI-REML algorithm for the standard model have been given by [11, 12] for univariate and [13, 14] for multivariate analyses. In brief, the likelihood for Eq. (1) and its first derivatives, written in terms of its MME (Eq. 2), are:

7 $$-2\, \log {\varvec{\mathcal {L}}}= const + \log \left| \mathbf{R} \right| + \log \left| \mathbf{H} \otimes {\varvec{\Sigma}_{u}}\right| + \log \left| \mathbf{C} \right| + \mathbf{y}^{\prime} \mathbf{Py},$$

and

8 $$-2\ \frac{\partial \log {\varvec{\mathcal {L}}}}{\partial \theta _i}= \frac{\partial \log \left| \mathbf{R} \right|}{\partial \theta _i}+ \frac{\partial \log \left| \mathbf{H} \otimes {\varvec{\Sigma}_{u}}\right|}{\partial \theta _i} + \frac{\partial \log \left| \mathbf{C} \right|}{\partial \theta _i} + \frac{\partial \mathbf{y}^{\prime} \mathbf{Py} }{\partial \theta _i}$$

9 $$= {\text{tr}}\left( \mathbf{R}^{-1}\frac{\partial \mathbf{R}}{\partial \theta _i}\right) + N {\text{tr}}\left( {\varvec{\Sigma}}_{u}^{-1} \frac{\partial {\varvec{\Sigma}_{u}}}{\partial \theta _i} \right) + {\text{tr}}\left( \mathbf{C}^{-1} \frac{\partial \mathbf{C}}{\partial \theta _i} \right) + \mathbf{y}^{\prime} \mathbf{P} \frac{\partial \mathbf{V}}{\partial \theta _i} \mathbf{P} \mathbf{y} ,$$

where ${\text{tr}}$ denotes the matrix trace operator. The ‘data part’ of $\log {\varvec{\mathcal {L}}}$ is the quadratic in the vector of observations, $\mathbf{y}^{\prime} \mathbf{P} \mathbf{y}$, where $\mathbf{P}=\mathbf{V}^{-1}- \mathbf{V}^{-1}\mathbf{X} \left( \mathbf{X}^{\prime} \mathbf{V}^{-1}\mathbf{X}\right) ^{-} \mathbf{X}^{\prime} \mathbf{V}^{-1}$ with $\mathbf{V} = \mathbf{Z} \left ( \mathbf{H} \otimes {\varvec{\Sigma}_{u}} \right ) \mathbf{Z}^{\prime} + \mathbf{R}$. Hence the elements of the average information matrix are proportional to [11]:

10 $$\frac{\partial ^2 \mathbf{y}^{\prime} \mathbf{P} \mathbf{y}}{\partial \theta _i \partial \theta _j}= \mathbf{y}^{\prime} \mathbf{P} \frac{\partial \mathbf{V}}{\partial \theta _i} \mathbf{P} \frac{\partial \mathbf{V}}{\partial \theta _j} \mathbf{P} \mathbf{y} .$$

Corresponding quantities for the full rank PC model are [10]:

11 $$-2\, \log {\varvec{\mathcal {L}}}= const + \log \left| \mathbf{R} \right| + \log \left| \mathbf{H} \otimes \mathbf{I}_q\right| + \log \left| \mathbf{C}^{\star}\right| + \mathbf{y}^{\prime} \mathbf{P}^{\star}\mathbf{y}$$

12 $$-2\, \frac{\partial \log {\varvec{\mathcal {L}}}}{\partial \theta _i}= {\text{tr}}\left( \mathbf{R}^{-1}\frac{\partial \mathbf{R}}{\partial \theta _i}\right) + {\text{tr}}\left( \mathbf{C}^{\star -1}\frac{\partial \mathbf{C}^{\star}}{\partial \theta _i} \right) + \mathbf{y}^{\prime} \mathbf{P}^{\star}\frac{\partial \mathbf{V}^{\star}}{\partial \theta _i} \mathbf{P}^{\star}\mathbf{y}$$

13 $$\frac{\partial ^2 \mathbf{y}^{\prime} \mathbf{P}^{\star}\mathbf{y}}{\partial \theta _i \partial \theta _j}= \mathbf{y}^{\prime} \mathbf{P}^{\star}\frac{\partial \mathbf{V}^{\star}}{\partial \theta _i} \mathbf{P}^{\star}\frac{\partial \mathbf{V}^{\star}}{\partial \theta _j} \mathbf{P}^{\star}\mathbf{y} ,$$

with $\mathbf{V}^{\star}= \mathbf{Z}^{\star}\left ( \mathbf{H} \otimes \mathbf{I}_q \right ) \mathbf{Z}^{\star}{^{\prime}} + \mathbf{R}$ and $\mathbf{P}^{\star}$ as **P** but with $\mathbf{V}^{\star}$ replacing **V**. Note that there are no explicit contributions involving ${\varvec{\Sigma}_{u}}$ in Eqs. (11) and (12) as these are incorporated into $\mathbf{C}^{\star}$.

Derivatives of $\mathbf{y}^{\prime}\mathbf{P}^{\star}\mathbf{y}$ can be obtained without forming $\mathbf{P}^{\star}$ or $\mathbf{V}^{\star -1}$ explicitly. Selected details for the first derivatives are given in “Appendix” and computing strategies to evaluate second derivatives are described by [10–13, 18].

Early implementations of multivariate REML analyses applied a parameterisation to estimate the $q(q+1)/2$ distinct elements of each covariance matrix to be estimated, ${\varvec{\Sigma}_{x}}$, directly. However this proved problematic when employing Newton(-Raphson) type algorithms (Eq. 6) to maximise $\log {\varvec{\mathcal {L}}}$ as these did not guarantee estimates to be within the parameter space. Reparameterising to the elements of the Cholesky factors of the ${\varvec{\Sigma}_{x}}$ teamed with taking logarithms of the diagonal elements resolved this problem and, moreover has been reported to yield good convergence rates [19, 20].

## Material and methods

### Data

We contrast sparsity patterns of the coefficient matrix and the resulting computational requirements for REML estimation for the MV and the PC parameterisation for two data sets. As only the structure of the resulting MME is of importance, only selected details are reported in the following.

Data set 1 included data for five correlated traits recorded on 21,000 individuals in eight generations, simulated using the software package AlphaSim, version 1.05 [21]. There were 2100 and 3150 animals in generations 1 to 4 and 5 to 8 which were progeny of 100 and 150 sires and 1000 and 1500 dams, respectively. To mimic a distribution over small, fixed effects subclasses, records were randomly assigned to 301 ‘contemporary groups’ per generation. Genotypes were constructed by sampling 125 quantitative trait loci and 32,000 single nucleotide polymorphisms (SNPs). Only marker information for 10%, 30%, 40% and 50% of randomly chosen individuals in generations 5 to 8 was retained. This yielded 4121 genotyped and 16,879 non-genotyped animals.

Data set 2 consisted of data for four phenotypes recorded on sheep. There were 87,369 animals in the data with 81,130, 71,852, 51,628 and 7692 records for traits 1 to 4, distributed over 4823, 6085, 4857 and 945 contemporary groups, respectively. Genotype information (33,887 markers) was available for 6112 individuals out of 107,730 animals in the pedigree.

### Analyses

Genomic relationship matrices (**G**) were built using Method 1 of Van Raden [22], eliminating SNPs with minor allele frequencies lower than 2% and centering allele counts using ‘observed’ frequencies. **G** was aligned with $\mathbf{A}_{22}$, the submatrix of the numerator relationship matrix for genotyped animals, as described by Vitezica et al. [23]. This was used to set up $\mathbf{H}^{-1}$, the inverse of the joint relationship matrix for genotyped and non-genotyped individuals [1].

Uni- and multivariate REML analyses for both parameterisations were carried out considering traits 1 to *t* for $t=1,\ldots , q$. Analyses employed the AI-REML algorithm [12], using a supernodal approach [24] to factor and invert **C** or $\mathbf{C}^{\star}$. Here the term ‘supernodal’ describes a computing strategy which identifies dense diagonal blocks of the matrix and carries out the calculations required block-wise rather than row by row which allows exploitation of highly optimised (and parallelised) library routines. Analyses were parameterised to estimate the elements of the Cholesky factors of the covariance matrices due to residuals and random effects fitted. Elapsed or ‘wall’ times were recorded for the set-up phase which included establishing a fill-in reducing reordering for the Cholesky factorisation of the coefficient matrix using an approximate minimum degree algorithm [25], symbolic factorisation and the first likelihood evaluation, but excluded the time needed to compute $\mathbf{H}^{-1}$. Times per iterate were obtained as the average over three or four AI-REML iterates which each involved a single likelihood evaluation only.

Analyses were carried out considering both the inverse of the joint relationship matrix and its counterpart based on pedigree information only ($\mathbf{A}^{-1}$). For data set 1, a simple animal model was fitted. This included either overall means or contemporary groups as the only fixed effects. For data set 2, a simple animal model (including contemporary groups as fixed effects) was contrasted with a model fitting both genetic (107,330) and permanent environmental maternal (20,487) effects as additional random effects. For this, direct and maternal genetic effects were treated as uncorrelated but assumed to have the same relationship matrix.

Computations were carried out on a desktop computer running Linux, fitted with an Intel I7-7820X processor with 8 cores rated at 3.6 Ghz and 64 GB of RAM. REML analyses were performed using our software package $\mathbb {WOMBAT}$ [16, 17] using up to 8 threads.

## Results and discussion

Elapsed times for data set 1 are summarised in Table 1. As observed in previous investigations (unpublished), there was no discernible difference in execution times between MV and PC when using pedigree derived relationships only. With 8,564,005 non-zero elements in $\mathbf{H}^{-1}$ (single triangle) to be processed  — compared to 77,616 in $\mathbf{A}^{-1}$ – computing times required for ssGREML analyses were higher by orders of magnitude, more so than reported by [3]. In this scenario, however, the PC model performed consistently better both in terms of setup times and times per iterate and the more so the larger the number of traits considered and the less fixed levels were fitted.

**Table 1:** Elapsed times (seconds) for analyses of the simulated data

		Means only					Contemporary groups				
	$t^{\text{a}}$	1	2	3	4	5	1	2	3	4	5
NEQ^b^		21,001	42,002	63,003	84,004	105,005	23,408	46,816	70,224	93,632	117,040
Pedigree relationships only											
Setup^c^	MV^d^	1	3	6	9	13	2	6	12	22	33
	PC^e^	1	3	6	9	14	2	6	12	22	33
Iterate^f^	MV	< 1	1	2	5	8	1	7	19	39	74
	PC	< 1	1	2	5	9	1	7	19	40	75
Pedigree and genomic relationships											
Setup	MV	28	195	636	1459	2,836	30	203	643	1502	2896
	PC	28	71	141	225	321	32	81	154	251	374
Iterate	MV	14	206	1209	4424	12,357	19	243	1282	4637	12,853
	PC	8	34	87	171	292	12	56	134	262	549
	R^g^	1.8	6.1	13.9	25.9	42.3	1.6	4.3	9.6	17.7	28.0
NZ-**C**^h^	MV	8.61	34.36	77.27	137.32	214.52	8.61	34.37	77.29	137.35	214.57
	PC	8.61	17.28	26.00	34.80	43.66	8.61	17.29	26.03	34.84	43.71
NZ-**L**^i^	MV	11.77	47.02	105.74	187.93	293.61	21.06	83.98	188.91	335.79	524.63
	PC	11.77	46.98	105.63	187.72	293.25	21.06	83.94	188.78	335.54	524.22

^a^Number of traits considered

^b^Number of equations in mixed model

^c^Time for set-up steps of REML analysis

^d^Standard multivariate parameterisation

^e^Principal components parameterisation

^f^Time per AI-REML iterate

^g^Ratio of times per iterate: MV/PC

^h^Number of non-zero elements in one triangle of the coefficient matrix; in millions

^i^Number of non-zero elements in the Cholesky factor of the coefficient matrix; in millions

Corresponding results for data set 2, together with the characteristics of the coefficient matrix in the MME, are in Table 2. Overall, timings exhibited a similar pattern to that found for data set 1, although, with a much smaller proportion of genotyped animals, ratios of times per iterate for MV over PC were somewhat lower (but still very worthwhile), especially for the analyses fitting maternal effects. Similarly with many contemporary groups fitted as fixed effects, set-up times for PC and MV differed much less than for the simulated data.

**Table 2:** Elapsed times (seconds) together with the characteristics of the coefficient matrix in the mixed model equations for analyses of sheep data

		Simple animal model				Fitting maternal effects			
	*t*^a^	1	2	3	4	1	2	3	4
NP^b^		2	6	12	20	4	12	24	40
NEQ^c^		112,533	226,368	338,955	447,630	239,309	482,364	723,606	960,498
Pedigree relationships only									
Setup^d^	MV^e^	14	33	57	77	28	88	176	255
	PC^f^	14	34	65	100	28	95	206	358
Iterate^g^	MV	6	17	31	46	13	47	100	171
	PC	6	17	31	45	13	46	97	176
Pedigree and genomic relationships									
Setup	MV	107	660	2093	4860	223	1290	4140	9158
	PC	107	587	1784	3996	223	1137	3298	7126
Iterate	MV	52	644	3620	13,250	157	1783	9965	35,298
	PC	33	121	278	497	109	405	940	1905
	R^h^	1.6	5.4	13.0	26.7	1.4	4.4	10.6	18.5
NZ-**C**^i^	MV	19.2	76.4	171.6	304.5	38.6	153.6	344.7	610.6
	PC	19.2	38.6	58.0	77.1	38.6	77.9	117.5	155.9
NZ-**L**^j^	MV	29.2	109.2	249.0	437.5	86.2	346.9	781.3	1343.6
	PC	29.2	111.6	249.8	421.3	86.2	345.5	747.5	1290.4

^a^Number of traits considered

^b^Number of parameters to be estimated

^c^Number of equations in mixed model

^d^Time for set-up steps of REML analysis

^e^Standard multivariate parameterisation

^f^Principal components parameterisation

^g^Time per AI-REML iterate

^h^Ratio of times per iterate: MV/PC

^i^Number of non-zero elements in one triangle of the coefficient matrix; in millions

^j^Number of non-zero elements in the Cholesky factor of the coefficient matrix; in millions

Differences in the numbers of non-zero elements in the coefficient matrices (NZ-**C** in Tables 1 and 2) for MV and PC, **C** and $\mathbf{C}^{\star}$ respectively, increased markedly with the number of traits considered, highlighting their differences in sparsity. This was due to elements of $\mathbf{H}^{-1}$ contributing only *q* non-zero coefficients each rather than $q^2$; see Eq. (5) versus Eq. (3). As demonstrated earlier [15], the latter can markedly reduce the computational burden for multivariate ssGBLUP analyses where the MME are held in core.

However, during factorisation of these matrices, substantial numbers of new elements arose, commonly known as ‘fill-in’. Hence, the numbers of non-zero elements in the Cholesky factors of **C** and $\mathbf{C}^{\star}$ – and thus the peak memory required — did no longer differ dramatically (NZ-**L** in Tables 1 and 2). Moreover, this translated directly to the subset of elements of their inverses which needed to be calculated, and thus only contributed to a relatively small extent to the reduction in execution time per AI-REML iterate for PC over MV.

Each iterate of AI-REML requires calculation of the partial, first derivative of the likelihood function for each of the parameters $\theta _i$ to be estimated. Among other parts, this involves evaluation of ${\text{tr}}\left ( \mathbf{C}^{-1} \, [\partial \mathbf{C}/\partial \theta _i] \right )$ (MV) or ${\text{tr}}\left (\mathbf{C}^{\star -1}[ \partial \mathbf{C}^{\star}/\partial \theta _i] \right )$ (PC); see Eqs. (9) and (12). For either model, this requires the inverse of the coefficient matrix which can be computationally demanding. An alternative used earlier was based on ‘automatic differentiation’ of the Cholesky factor of the coefficient matrix [20]. However, this was found to be no longer competitive when sparse matrix inversion was adapted to a supernodal approach and multi-threaded execution (unpublished) and is thus no longer recommended.

The formulation above implies that computing times for the first derivatives depend on the sparsity pattern of the derivatives of the coefficient matrix. This holds in particular for the $q(q+1)/2$ parameters modelling ${\varvec{\Sigma}_{u}}$, $\theta _{ui}$. For MV, $\partial \mathbf{C}/ \partial \theta _{ui} = \mathbf{H}^{-1}\otimes \partial {\varvec{\Sigma}}_{U}^{-1}/ \partial \theta _{Ui}$, i.e. only the second part of $\mathbf{C}_{uu}$ contributes non-zero coefficients and computations required are directly determined by the number of non-zero elements of $\mathbf{H}^{-1}$. In contrast, for PC, submatrices $\mathbf{C}_{bu}^{\star}$ and the first part of $\mathbf{C}_{uu}^{\star}$ contribute non-zero elements to $\partial \mathbf{C}^{\star}/ \partial \theta _{ui}$. As outlined above, the latter — $\mathbf{Z}^{\star}{^{\prime}} \mathbf{R}^{-1}\mathbf{Z}^{\star}$ — is blockdiagonal for animals with blocks of size $q \times q$ (assuming that the levels of **u** are ordered by traits within animals). Moreover, the second part of $\mathbf{C}_{uu}^{\star}$, $\mathbf{H}^{-1}\otimes \mathbf{I}_q$, does not depend on the parameters to be estimated and thus its derivatives with respect to $\theta _{ui}$ are zero. This means that computations for the calculation of the derivatives do not depend on the structure of $\mathbf{H}^{-1}$, which explains the substantial reductions in elapsed time per iterate observed for the PC model. Corresponding arguments hold for the second component of the first derivatives that involves the coefficient matrix of the MME, namely $\partial \mathbf{y}^{\prime}\mathbf{P}\mathbf{y}/ \partial \theta _{ui}$ versus $\partial \mathbf{y}^{\prime}\mathbf{P}^{\star}\mathbf{y}/ \partial \theta _{ui}$ (see “Appendix”).

## Conclusions

We have demonstrated that a simple reparameterisation of the mixed model fitted can result in substantially reduced computing times for ssGREML analyses, i.e. REML analyses accounting for genomic relationships or involving similar, partially dense relationship matrices. It should be noted that this does not affect convergence behaviour, yielding the same estimates and changes in likelihood in each AI-REML iterate for both the PC and standard MV scale.

Clearly, the timings presented are highly implementation-specific. The REML software used did not provide any specific provisions to account for dense parts in $\mathbf{H}^{-1}$, i.e. dealt with $\mathbf{H}^{-1}$ element by element. Computing times could be improved, especially for the MV parameterisation, by arranging calculations so that the submatrix of $\mathbf{H}^{-1}$ for genotyped animals is held in a block, allowing it to be processed using highly optimised library routines available for dense matrix calculations. Other possible refinements of sparse matrix storage schemes are outlined, for instance, by Masuda et al. [3] and are likely to facilitate further improvements. Such measures may reduce the advantage of the PC model over the MV model. Nevertheless, the equivalent parameterisation described here extends the range of ssGREML analyses that are computationally readily feasible and thus provides a useful addition to our armoury for quantitative genetic analyses in the genomic age.

## Data Availability

The simulated data set used in this study is available from the corresponding author on reasonable request.

## Appendix

For MV, $\mathbf{Py} = \mathbf{R}^{-1} \hat{\mathbf{e}}$ with $\hat{\mathbf{e}} = \mathbf{y} - \mathbf{X} \hat{{\varvec{\upbeta}}} - \mathbf{Z} \hat{\mathbf{u}}$ and $\mathbf{Z}^{\prime} \mathbf{P} \mathbf{y} = (\mathbf{H}^{-1}\otimes {\varvec{\Sigma}}_{u}^{-1}) \hat{\mathbf{u}}$ [11]. This gives first derivatives (see also [13]):

14 $$\frac{\partial \mathbf{y}^{\prime}\mathbf{P}\mathbf{y}}{\partial \theta _{ui}}= \hat{\mathbf{u}}^{\prime}\left( \mathbf{H}^{-1}\otimes {\varvec{\Sigma}}_{u}^{-1} \frac{\partial {\varvec{\Sigma}_{u}}}{\partial \theta _{ui}} {\varvec{\Sigma}}_{u}^{-1} \right) \hat{\mathbf{u}}$$

15 $$\frac{\partial \mathbf{y}^{\prime}\mathbf{P}\mathbf{y}}{\partial \theta _{ei}} = \hat{\mathbf{e}}^{\prime} \mathbf{R}^{-1}\frac{\partial \mathbf{R}}{\partial \theta _{ei}} \mathbf{R}^{-1}\hat{\mathbf{e}} .$$

Corresponding terms on the PC scale are:

16 $$\frac{\partial \mathbf{y}^{\prime} \mathbf{P}^{\star}\mathbf{y}}{\partial \theta _{ui}}= 2\, \hat{\mathbf{e}}^{\star}{^{\prime}} \mathbf{R}^{-1}\frac{\partial \mathbf{Z}^{\star}}{\partial \theta _{ui}} \hat{\mathbf{u}}^{\star}\quad \text {with} \quad \hat{\mathbf{e}}^{\star}= \mathbf{y} - \mathbf{X} \hat{{\varvec{\upbeta}}} - \mathbf{Z}^{\star}\hat{\mathbf{u}}^{\star}$$

17 $$\frac{\partial \mathbf{y}^{\prime}\mathbf{P}^{\star}\mathbf{y}}{\partial \theta _{ei}}= \hat{\mathbf{e}}^{\star}{^{\prime}} \mathbf{R}^{-1}\frac{\partial \mathbf{R}}{\partial \theta _{ei}} \mathbf{R}^{-1}\hat{\mathbf{e}}^{\star}.$$

It can be shown that:

18 $$- \left[\begin{array}{ll} \hat{{\varvec{\upbeta}}}^{\prime} \vdots \, \hat{\mathbf{u}}^{\star}{^{\prime}} \end{array}\right] \frac{\partial \mathbf{C}^{\star}}{\partial \theta _{ui}} \left[\begin{array}{ll} \hat{{\varvec{\upbeta}}}\\ \hat{\mathbf{u}}^{\star}\end{array}\right]= 2\, \hat{\mathbf{e}}^{\star}{^{\prime}} \mathbf{R}^{-1}\frac{\partial \mathbf{Z}^{\star}}{\partial \theta _{ui}} \hat{\mathbf{u}}^{\star}-2 \, \mathbf{y}^{\prime} \mathbf{R}^{-1}\frac{\partial \mathbf{Z}^{\star}}{\partial \theta _{ui}} \hat{\mathbf{u}}^{\star},$$

i.e. that the derivatives of $\mathbf{y}^{\prime}\mathbf{P}^{\star}\mathbf{y}$ with respect to $\theta _{ui}$ can conveniently be evaluated alongside the respective terms ${\text{tr}}\left ( [\partial \mathbf{C}^{\star}/ \partial \theta _{ui}] \mathbf{C}^{\star -1}\right )$ required in Eq. (12).
